# Three steps to reduction surgical site infection: presentation of a comprehensive model

**DOI:** 10.3205/dgkh000443

**Published:** 2023-08-16

**Authors:** Saeid Amini Rarani, Axel Kramer

**Affiliations:** 1 Department of Operating Room, Nursing and Midwifery Care Research Center, Isfahan University of Medical Sciences, Isfahan, Iran; 2 Institute of Hygiene and Environmental Medicine, University Medicine Greifswald, Greifswald, Germany

**Keywords:** surgical site infections, prevention, perioperative measures, multidisciplinary collaboration, continuous quality improvement

## Abstract

To prevent surgical site infections (SSIs), a three-step model is proposed, which integrates perioperative measures, multidisciplinary collaboration, and continuous quality improvement (CQI) initiatives.

## Abstract (de)

Zur Prävention von postoperativen Wundinfektionen wird ein 3-Stufen Modell vorgeschlagen, in dem perioperative Maßnahmen, multidisziplinäre Zusammenarbeit und kontinuierliche Qualitätsverbesserung aufeinander abgestimmt umgesetzt werden.

## Introduction

Surgical site infections (SSIs) are defined as infections occurring up to 30 days after surgery (or up to one year after surgery in patients receiving implants) and affecting either the incision or deep tissue at the surgical site [1]. SSIs are a significant concern, as they not only lead to increased morbidity and mortality rates but also impose a considerable economic burden on healthcare systems worldwide. SSIs pose a substantial challenge to patient safety and healthcare systems worldwide. Despite advancements in surgical techniques and infection control measures, SSIs continue to occur at an alarming rate. SSIs are responsible for approximately $3.5 billion to $10 billion in US healthcare costs annually. Compared to patients without SSIs, those with SSIs remain in the hospital approximately 7 to 11 days longer; one study involving 177,706 postsurgical patients reported that SSI was the cause of 78% of all readmissions [2]. Therefore, measures to reduce surgical site infection are necessary [3] and must be adaptedunder pandemic situations [4].

## Results

The proposed 3-step model integrates perioperative measures, multidisciplinary collaboration, and continuous quality improvement (CQI) initiatives to enhance patient safety and outcomes.

### Perioperative measures 

This subsection emphasizes the importance of adhering to evidence-based guidelines for infection prevention during surgical procedures. It explores preoperative strategies (e.g., patient optimization, antimicrobial prophylaxis), intraoperative measures (e.g., sterile technique adherence), and postoperative care (e.g., wound management) [1].

#### Preoperative strategies 

##### Patient optimization 

Preoperatively identifying and addressing modifiable risk factors such as obesity, smoking cessation programs, glycemic control in diabetic patients, and decolonization of nasal carriers of Staphylococcus aureus can significantly reduce the risk of SSIs.

##### Antimicrobial prophylaxis 

Administering appropriate antimicrobial prophylaxis based on established guidelines is crucial in preventing SSIs.

##### Skin preparation 

Proper skin preparation using residually-acting alcohol-based formulations that contain e.g. povidone-iodine or chlorhexidine gluconate, significantly reduces bacterial colonization at the surgical site.

#### Intraoperative strategies 

##### Aseptic technique 

Adherence to strict aseptic techniques during surgery minimizes contamination risks.

##### Surgical attire 

Wearing appropriate surgical attire including sterile gowns and gloves reduces the introduction of pathogens into the surgical field [5].

##### Surgical drapes 

The use of sterile surgical drapes creates a barrier between the surgical site and potential sources of contamination.

##### Surgical site irrigation 

Antiseptic irrigation solutions, such as polyhexanide or povidone-iodine, can be used intraoperatively to reduce bacterial load at the surgical site.

#### Postoperative strategies 

##### Wound care 

Proper wound care techniques, including regular dressing changes and monitoring for signs of infection, are essential in preventing SSIs.

##### Early mobilization 

Encouraging early mobilization postoperatively aids in improving blood circulation and reducing the risk of infection.

##### Antibiotic stewardship 

Rational use of antibiotics, including appropriate duration and selection based on culture results, helps prevent the development of antibiotic-resistant organisms.

### Multidisciplinary collaboration

#### The role of surgeons 

Surgeons play a pivotal role in preventing SSIs, for instance, through appropriate antimicrobial prophylaxis, meticulous surgical technique, and timely wound closure. Collaborative efforts with other healthcare professionals can enhance compliance with these practices and promote standardized protocols.

#### The role of nurses 

Nurses are integral members of the multidisciplinary team involved in perioperative care. Their contributions include preoperative patient education, strict adherence to aseptic techniques during surgery, effective wound management postoperatively, and surveillance for early detection of SSIs.

#### The role of anesthesiologists 

Anesthesia providers contribute significantly to SSI reduction by optimizing patients’ physiological status before surgery, ensuring normothermia during procedures, and implementing strategies such as antibiotic stewardship programs that minimize the risk of infection.

#### Infection prevention specialists 

Infection prevention specialists play a critical role in developing comprehensive infection control programs tailored to each surgical setting’s unique needs. Their involvement includes surveillance, monitoring compliance with infection control practices, and providing education and training to healthcare professionals.

#### Importance of environmental services 

Environmental services personnel play a crucial role in maintaining a clean and hygienic surgical environment. Their collaboration with the multidisciplinary team ensures proper cleaning and disinfection surfaces and validated reprocessing of surgical equipment.

#### The role of information technology 

Information technology systems can facilitate communication, enhance data collection, and support decision-making processes related to SSI prevention. Collaborative efforts between healthcare professionals and IT specialists can lead to the development of innovative tools for real-time monitoring, risk assessment, intervention implementation and auditing [3].

#### Patient engagement 

Engaging patients in their care is vital for SSI reduction. Educating patients about preoperative hygiene measures, promoting adherence to prescribed medications, and involving them in shared decision-making processes can empower patients to actively participate in infection prevention.

### CQI initiatives 

To sustain long-term improvements in SSI rates, this subsection emphasizes the importance of CQI initiatives. It explores the use of surveillance systems, data analysis, feedback mechanisms, and regular audits to identify areas for improvement and implement targeted interventions.

#### Methodologies employed in CQI initiatives 

Various methodologies are utilized in CQI initiatives to reduce SSIs. This section explores the application of Lean Six Sigma principles, Plan-Do-Study-Act cycles, root cause analysis, process mapping, and other quality improvement tools in different healthcare settings.

#### Outcomes of CQI initiatives 

This section presents a comprehensive analysis of the outcomes associated with CQI initiatives aimed at reducing SSIs. It examines reductions in SSI rates, improvements in compliance with infection prevention protocols, enhanced patient satisfaction scores, decreased length of hospital stays, and cost savings achieved through these initiatives.

## Conclusions

In conclusion, SSIs remain a significant concern in operating rooms worldwide. However, by implementing a comprehensive model that integrates perioperative measures, multidisciplinary collaboration, and CQI initiatives, healthcare institutions can effectively decrease the incidence of SSIs and improve patient outcomes.

## Notes

### Competing interests

The authors declare that they have no competing interests.

### Authors’ ORCIDs:

- Saeid Amini Rarani: 0000-0003-4700-0211
- Axel Kramer: 0000-0003-4193-2149
